# The cryo-EM resolution revolution and transcription complexes

**DOI:** 10.1016/j.sbi.2018.07.002

**Published:** 2018-10

**Authors:** Jonas Hanske, Yashar Sadian, Christoph W Müller

**Affiliations:** European Molecular Biology Laboratory (EMBL), Structural and Computational Biology Unit, Meyerhofstraße 1, 69117 Heidelberg, Germany

## Abstract

Direct electron detector technology combined with improved imaging processing procedures has dramatically increased the resolution that can be obtained by single-particle cryo-electron microscopy and cryo-electron tomography. These developments — often referred to as the `resolution revolution’ in cryo-EM — have had a profound impact on the structural biology of transcription as they allow the determination of atomic or near-atomic resolution structures of very large, flexible and often transient transcription complexes that in many cases had resisted crystal structure determination for decades. In this review, we will discuss recent advances and breakthroughs in the structural biology of transcription complexes enabled by the revolution in cryo-electron microscopy with particular focus on eukaryotic RNA polymerases and their pre-initiation complexes, but also chromatin remodelers and epigenetic regulators.

## Highlights

• Gold rush in cryo-EM structure determination of challenging transcription complexes.
• Initiation complexes of all eukaryotic RNA polymerases have been solved by cryo-EM.
• Cryo-EM structures of chromatin modifiers become available at increasing pace.

## Graphical abstract

**Figure:**
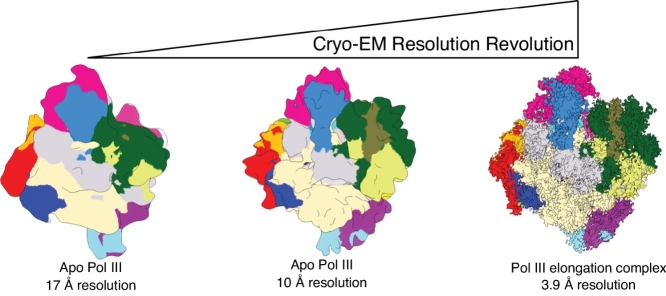

**Current Opinion in Structural Biology** 2018, **52**:8–15

This review comes from a themed issue on **Cryo electron microscopy**

Edited by **John Briggs** and **Werner Kuhlbrandt**

For a complete overview see the Issue and the Editorial

Available online 14th July 2018

**https://doi.org/10.1016/j.sbi.2018.07.002**

0959-440X/© 2018 The Authors. Published by Elsevier Ltd. This is an open access article under the CC BY license (http://creativecommons.org/licenses/by/4.0/).

## Introduction

Gene transcription is a fundamental process of life performed by a set of highly dynamic molecular machines with RNA polymerase as a core component. Transcriptional activity is cell-type specific and undergoes tremendous changes during the cell cycle. As such, tight control over the activity of the transcription machinery is required [1]. In eukaryotes, transcriptional regulation occurs at the level of the three eukaryotic RNA polymerases, associated general and sequence-specific transcription factors, and co-activators such as Mediator. On a second level, chromatin modifying and chromatin remodeling complexes regulate the accessibility of chromatin for transcription.

**Figure 1:**
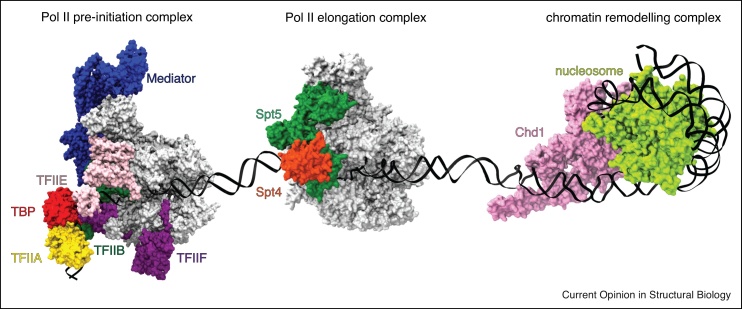
Landscape of Pol II-mediated transcription. Steps of the transcription cycle from formation of pre-initiation complex (left) to elongation (middle), preceded by nucleosome removal by chromatin remodeler Chd1 (right) is depicted. In the PIC (pdb: 5fyw), Pol II is shown in grey, TBP in red, TFIIA in yellow, TFIIB in dark green, TFIIF in purple, TFIIE in pink, and Mediator in blue. In the Pol II elongation complex (pdb: 5oik), Pol II is depicted in grey, Spt5 in green and Spt4 in orange. In the nucleosome–Chd1 complex (pdb: 5o9g), the nucleosome and Chd1 are colored in light green and magenta, respectively. DNA is colored in black.

During the last decades, X-ray crystallography has been the central structural biology technique, providing a constant flow of molecular structures which gave important insight into eukaryotic transcription in the context of chromatin (see also the 100 landmark crystal structures that influenced transcription and chromatin research [2]). Despite these enormous successes, transcription complexes are nevertheless challenging targets for X-ray crystallography not only due to their size (often there are several dozens of polypeptide chains involved), but also because of their inherent flexibility, their modular structure, the transient interactions of their components and their limited accessibility [3]. Accordingly, the crystallization and X-ray structure determination of large transcription complexes is often a painstakingly long but also risky process that is not always crowned by success. During the last years, single-particle cryo-EM has increasingly gained importance as an alternative or complementary technique in the analysis of transcription complexes. Compared to X-ray crystallography, sample consumption is lower, larger complexes are more easily observed, and conformational heterogeneity can be overcome by particle classification in many cases. For many years, progress in cryo-EM was halted by the low resolution of the structures obtained due to technical limitations such as detector sensitivity. However, recent technical advances such as direct electron detectors combined with improved single-particle cryo-EM data acquisition and data processing pipelines, coined as `the resolution revolution’ [4], have caused a gold rush in the transcription field with a large number of transcription complex structures having become available only within the last few years (Figure 1). Here, we highlight the impact of the cryo-EM resolution revolution in the field of transcription and chromatin research and review recent insights on eukaryotic RNA polymerase transcription complexes and chromatin modifier complexes obtained by cryo-EM.

## Transcription initiation of eukaryotic polymerases

In eukaryotes, three different RNA polymerases (Pol I, II, and III) catalyze DNA-dependent transcription into distinct RNA products by the same conserved mechanism also present in bacteria and archaea [5]. However, eukaryotes have evolved a large and diverse set of specific protein factors to ensure regulated promoter-dependent transcription initiation, elongation and termination. To initiate transcription, general transcription factors (GTF) recognize the promoter with the help of activators, assemble at the transcription start site, recruit their corresponding RNA polymerase to form a pre-initiation complex (PIC) and together facilitate DNA strand opening.

The crystal structure determination of the 10-subunit Pol II core enzyme and the Pol II elongation complex [6,7] were milestone achievements and served as starting point for the detailed structural analysis of Pol II transcription. At the time, the Pol II 10-subunit core structure was the largest asymmetric protein complex solved by X-ray crystallography. Moving beyond these initial crystal structures and obtaining Pol II crystal structures in different states and bound to transcription factors proved not to be an easy task. Obtaining the structure of the 12-subunit Pol II [8] and of elongating Pol II in complex with TFIIS [9] took an additional two years, while the complete complex of Pol II with TFIIB [10] was only solved six years later. Even larger assemblies of Pol II transcription complexes escaped crystallization attempts showing the limitations in resolving large and heterogeneous assemblies by X-ray crystallography due to the conformational and compositional heterogeneity of many transcription complexes.

At the time the Pol II crystal structure was solved, first cryo-EM structures of transcription complexes had already become available: TFIID together with TFIIA and TFIIB [11], TFIIH [12], and Mediator [13], an important co-activator of Pol II transcription. However, these structures generally suffered from low resolution limiting the biological conclusions that could be drawn from them. The limited resolution often also prevented accurate assignment of the cryo-EM densities and the unambiguous fitting of (partial) structures even in cases where crystal structures were available. However, the advent of direct electron detectors finally allowed overcoming this impediment.

In 2013, the Nogales lab proposed the structure of a minimal human Pol II PIC with cryo-EM map reconstructions at about 11 Å nominal resolution [14^••^], which was sufficient to confidently place homology models of Pol II and some GTFs. These results were followed shortly by intermediate resolution models of the yeast Pol II PICs [15,16] that, also supported by crosslinking mass-spectrometry [17], allowed the positioning of atomic models of many of the single components obtained by X-ray crystallography into the cryo-EM maps (reviewed in [18]). Finally, cryo-EM maps below 4.0 Å resolution enabled the construction of almost complete, near-atomic models of the human and yeast core Pol II PICs in different functional states [19,20].

Beyond the core Pol II PIC, several cryo-EM structures show the Pol II PIC bound to the Mediator complex [15,16,21], while structures of the multi-subunit GTFs TFIID and TFIIH were solved separately by cryo-EM [22,23]. Accordingly, we can now aim for super-assemblies and analyze them by cryo-EM as exemplified by the recent cryo-EM structure of the Pol II-TFIIH-mediator complex with a molecular mass of 2 MDa and comprising more than 46 polypeptides [24^••^] (Figure 1), while an even larger assembly that also comprises TFIID with a molecular mass of ∼3 MDa and >65 polypeptides at present only is a structural model that still awaits experimental validation [22]. It is obvious that the complexity and size of transcription complexes has reached a stage where X-ray crystallography is no longer the method of choice. However, in cryo-EM reconstructions the highest resolution is often only observed in the core, while more flexible peripheral subunits are observed at lower resolutions. Therefore, X-ray crystal structures often remain essential for the correct assignment as it has been also shown for the Mediator [24^••^,^25^,^26^].

Compared to the first high resolution 10-subunit Pol II core structure, atomic-resolution structures of 14-subunit Pol I and 17-subunit Pol III have only become available more than one decade later. This presumably reflects the additional difficulties in obtaining high-resolution crystal structures of both polymerases due to the additional subunits and the resulting increased flexibility. Shortly after the first crystal structures of apo Pol I could finally be obtained [27,28], structures of transcribing Pol I [29,30] and the promoter-binding competent Pol I [31,32] were also solved by cryo-EM, giving important further insight into its transcription mechanism. Only in the last year, three independent structures of the basal Pol I PIC comprising Pol I and core factor (CF) in an open complex were published [33^•^,34^•^,35^•^]. The crystal structure of CF obtained in one of the reconstructions [33^•^] greatly helped the complete interpretation of the cryo-EM maps. Although the structures generally agree on the overall architecture of the complex, certain flexible parts were differently positioned in the structures likely due to differences in the preparation.

**Figure 2:**
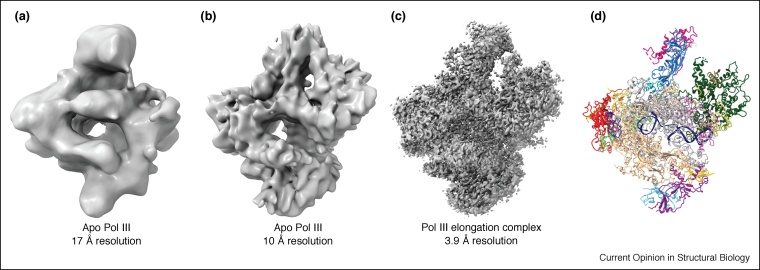
Resolution revolution in Pol III cryo-EM structures. **(a)** Electron density of apo RNA polymerase III obtained from particles recorded on Kodak SO163 films using a JEOL JEM2010 microscope at a magnification of 40 000× and processed with SPIDER to 17 Å (EMDB: 1322). **(b)** Electron density of apo RNA polymerase III recorded on Kodak SO163 films using a CM200 microscope at a magnification of 50 000× and processed with SPIDER to 10 Å (EMDB: 1804). **(c)** Electron density of the RNA polymerase III elongation complex recorded using a Titan Krios microscope equipped with Falcon II direct-electron detector at a magnification of 75 000× and processed using Relion to 3.9 Å (EMDB: 3178). **(d)** Model of the RNA polymerase III elongation complex (pdb: 5fj8) colored as in Hoffmann *et al*. [38].

In the case of the most complex eukaryotic RNA polymerase, 17-subunit Pol III, all attempts to solve the structure by X-ray crystallography were futile, while the structure elucidation of Pol III by cryo-EM gradually proceeded over almost 10 years paralleling the resolution revolution (Figure 2). The first cryo-EM structure of Pol III [36], resolved at 17 Å, already allowed appreciating the structural differences among the three eukaryotic polymerases and the approximate positions of the TFIIE-like and TFIIF-like subcomplexes, but the low resolution limited any further insight into the mechanism. An improved structural model resolved at 10 Å allowed for a better appreciation of the architecture of the Pol III-specific sub-complexes [37] and confirmed the high inherent flexibility of the peripheral sub-complexes. Finally, a `post-cryo-EM revolution’ Pol III structure at 3.9 Å resolution allowed for atomic model building of the entire complex [38]. The same preparation awarded three distinct conformational states: one of transcribing Pol III bound to a transcription scaffold and two apo Pol III structures both at slightly lower resolution. The increased resolution finally allowed visualizing individual side chains and this information can now be used for testing mechanistic hypothesis and to compare architecture and mechanisms of Pol I, Pol II and Pol III in detail.

Only this year, the Vannini group and our group independently proposed models for a minimal Pol III PIC [39^••^,40^••^]. Both groups used yeast Pol III together with TFIIIB bound to closed or artificially opened DNA constructs, which gave rise to distinct conformational states of the initially transcribing Pol III, and the PIC in closed and spontaneously opened complexes. In this case, the power of cryo-EM to catch distinct conformations in one sample preparation allowed for proposing a mechanism for DNA melting and OC formation in Pol III.

**Figure 3:**
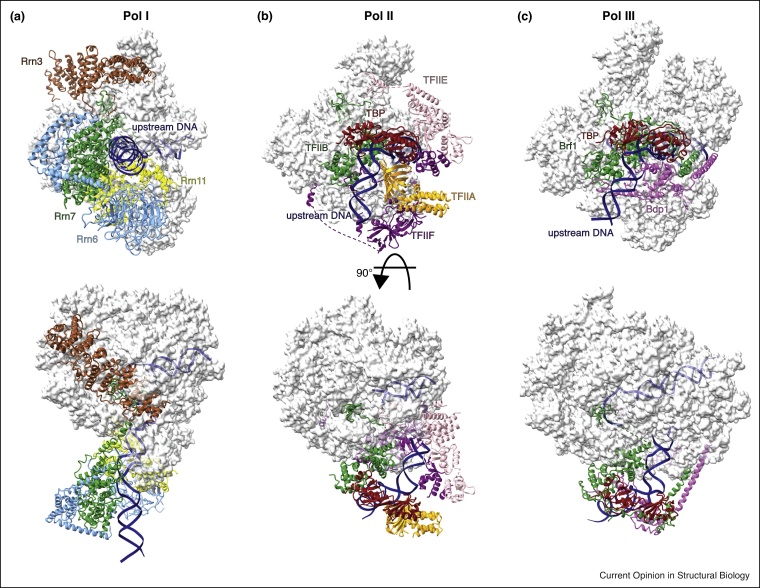
High resolution structures of RNA polymerase I, II and III pre-initiation complexes (PIC). **(a)** Pol I PIC (pdb: 5oa1) containing Pol I, Rrn3 (sienna) and Core Factor (CF) (Rrn6: blue, Rrn7 green, Rrn11: yellow) resolved at 4.4 Å [34^•^]. The path of upstream DNA is different compared to the Pol II and III PICs, and TFIIB-like Rrn7 is located further upstream from the polymerase as compared to TFIIB and Brf1. (**b)** Pol II PIC (pdb: 5fyw) containing Pol II, TFIIA (orange), TFIIB (green), TFIIE (pink), TFIIF (purple), and TBP (red) resolved at 3.6 Å [20]. **(c)** Pol III PIC (pdb: 6f40) containing Pol III and TFIIIB (Brf1, green; Bdp1, magenta; TBP, red) resolved at 3.7 Å [39^••^].

Comparing the recent PIC structures of all three eukaryotic RNA polymerases (Figure 3), a clear difference in architecture between Pol I PIC, on the one hand, and Pol II and Pol III PIC, on the other, can be appreciated. In the case of Pol II and Pol III, DNA follows a similar path and is translocated into the cleft of the polymerase upon opening, allowing the assumption that the general mechanism of DNA strand opening is conserved between Pol II and Pol III. In this process, the pre-assembled Pol III-specific subunits take over tasks that are performed by GTFs in the Pol II system thus providing also a rationale how Pol III achieves higher initiation rates than Pol II. In the case of Pol I, the highly divergent path of the upstream DNA in the open PIC suggests that Pol I translocates over the DNA by altering its position relative to the CF. This speculative mechanism rather resembles the way how bacterial polymerase achieves DNA promoter opening and might reflect the diverging complexity of regulation of transcription initiation in the three polymerases [41].

## Transcription elongation and termination

By contrast to transcription initiation, less structural information about the elongation complexes of eukaryotic RNA polymerases is available. In part, this is due to the transient nature of these complexes that involve the progressive synthesis of the RNA transcripts. The structure of transcribing yeast Pol II was solved by X-ray crystallography shortly after the Pol II apo enzyme [6,7] and the high degree of conservation among eukaryotes was demonstrated by a recent structure of the transcribing mammalian Pol II solved at 3.4 Å resolution [42]. There are also structures of elongating Pol I [29,30] and Pol III [38] solved by cryo-EM. However, in none of these structures elongation factors are present. Only last year, elongating Pol II was solved in complex with elongation factors (EFs) at high resolution [43^•^,^44^]. The structural model of Ehara *et al*. demonstrates how the basal EFs Elf1, Spt4/5, and TFIIS modify the polymerase surface thereby creating DNA exit and entry tunnels to keep the DNA in the active center thereby ensuring processive elongation. Nascent RNA is guided through another exit tunnel preventing interactions with single-stranded DNA that might lead to stalling. Recent attempts to obtain structural models of elongating Pol II together with Paf1C and TFIIS still suffer from low resolution [45] but there is little doubt that in the near future more structural models of elongation complexes will become available. This will likely be also the case for Pol I, which shares several EFs with Pol II [46]. Finally, Pol I, Pol II and Pol III use different mechanisms for transcription termination. Whereas Pol I and Pol II require binding of transcription termination factors, Pol III only requires a short stretch of 5 thymines on the non-template DNA strand as termination signal [47]. Considering the rapid elucidation of the other aspects of the transcription cycle in the recent years, we anticipate that cryo-EM structures will soon also contribute elucidating the transcription termination mechanisms of eukaryotic RNA polymerases.

## Chromatin remodelers, transcription activators, and Polycomb group proteins

To allow for promoter recognition, PIC assembly, and eventually transcription elongation, the DNA has to be made accessible from densely packed chromatin by displacing or shifting nucleosomes and recruiting GTFs and polymerases. Both nucleosomes and their regulators are challenging targets for structural biology due to their high inherent flexibility, their posttranslational modifications and the transient interactions of the complexes they form. After the X-ray crystal structure of the nucleosome core particle was solved some 20 years ago [48], it took almost two decades to obtain cryo-EM reconstructions of the same particle at a comparable resolution [49, 50, 51]. In addition to the limited resolution that was only overcome by the advent of direct electron detectors, progress was also slowed down by the preferred orientation of nucleosomes in the ice resulting in a limited number of independent views. With the cryo-EM nucleosome structure in hand, larger complexes comprising nucleosome-bound chromatin modifiers can now be tackled.

The chromatin-remodeling helicase-like ATPases enable transcription through active chromatin [52]. These multi-modular molecular machines modify chromatin architecture by acting on the nucleosome histone octamers, sliding them along DNA, altering their structure, and catalyzing their exchange or even their eviction [53]. Early work resulted only in low resolution structures that provided limited insight into the mode of action of these enzymes [54, 55, 56]. Only very recently, the structures of two chromatin remodelers bound to nucleosomes have been solved by cryo-EM with resolutions from 4.8 Å to just below 4 Å [57^•^,58^•^]. The structural model of yeast Snf2 bound to a nucleosome showed the resting state of the ATPase bound to DNA and interacting with the N-terminal tail of the histone H4 whereas the transition state of Chd-1 bound to DNA and the H4 tail was captured by using an ATP analog. Taken together, these data led to the proposal of a functional model of a ratcheting mechanism of the helicase translocation along the DNA thereby promoting histone sliding [58^•^]. Moreover, the structure of another chromatin remodeler, INO80, a large (>1 MDa) and flexible complex, was solved in its apo state by cryo-EM [59]. The high variation in resolution from 4.1 Å in the core to about 12 Å in the periphery reflects the high degree of flexibility of the complex. More recently, the architecture of the yeast NuA4/TIP60 complex has been solved at 4.7 Å, giving insight into the scaffolding and regulatory mechanism of this gene regulator and DNA repair enzyme complex [60].

Whereas chromatin remodelers actively regulate transcription in active chromatin, the family of Polycomb group (PcG) proteins control transcription on the epigenetic level by placing or removing epigenetic marks such as methyl groups or ubiquitin on histones to silence chromatin [61,62]. The overall architecture of the human Polycomb repressive complex 2 (PRC2), a highly modular and flexible protein complex, was first described by a negative-stain EM structure at 21 Å resolution [63]. Only very recently, more cryo-EM structures of PRC2 bound to its co-factors AEBP2 and JARID2 [64^••^] and of PRC2 bound to a di-nucleosome [65^••^] revealed how PRC2 is activated by a methylated lysine in histone H3 (H3 K27) in one nucleosome to methylate H3 K27 of the next nucleosome, thereby allowing methylation marks to spread. The interpretation of these cryo-EM reconstructions were greatly supported by the available crystal structures of a trimeric PRC2 core complex [66, 67, 68] demonstrating (again) the complementarity between crystal and cryo-EM structures. In yeast, the 19-subunit transcription co-activator complex SAGA is recruited to the transcription start site of Pol II by activators to alter the chromatin structure by modifying nucleosomal histones. Its structure has been elucidated with one of its central subunits, Tra1, resolved at medium resolution shedding light on the modular assembly of this major epigenetic regulator [69].

## Conclusions and perspectives

Cryo-EM has become an invaluable tool for the structural analysis of large transcriptional assemblies leading to a surge of publications within the last few years. Large transcription complexes such as the Pol II PIC and the PIC–core mediator complex [24^••^], RNA polymerase–elongation factor complexes, but also chromatin modifying complexes bound to nucleosomes whose size, conformational and compositional heterogeneity prevented crystallographic analysis can now be analyze by cryo-EM. At present most of these very large complexes are still determined at medium resolution and in many cases still rely on the availability of high-resolution X-ray structures of subunits and subcomplexes to obtain atomic structures. With the still increasing sensitivity of detectors, the introduction of the phase plate to allow for increased contrast at low defocus, improved sample preparation protocols, efforts to prevent surface denaturation of the sample using automatic sample dispenser like the Spotiton [70], and the advancement in computational analysis, we expect many high-resolution EM maps to be used for *ab initio* model building without prior knowledge of crystal structures.

One yet less explored application in the analysis of transcription complexes is time-resolved cryo-EM, which has enabled some recent breakthroughs in other biological applications [71]. The controlled mixing of complex components at millisecond timescales before plunge freezing can help capture more transient intermediate complexes for a more detailed understanding of the structural transitions between different conformational states.

All the structures discussed in this review were *in vitro* reconstitutions in many cases using recombinant subunits and complexes. Although *in vitro* systems offer a wealth of structural insight and lead to testable hypotheses, the vision is to observe these complexes also in their native environment, the nucleus. Recent advances in cryo-electron tomography (cryo-ET, reviewed in this issue) show promise that transcription complexes can be soon also observed in their cellular context. Cryo-ET has already allowed locating nucleosome chains [72] and lamin meshworks within the lamina [73], but hopefully soon will also picture specific transcription complexes in the nucleus such that high resolution single-particle cryo-EM structures can be positioned into the tomograms. However, preparing thin cellular samples remains challenging thus posing a serious limitation on the study of transcription complexes in their native environment. The coming years will bring more integrative studies utilizing these two methods. Nonetheless, functional studies in solution, both *in vitro* and *in vivo*, will still be required to test hypotheses derived from the structural data obtained by EM. Guided by the structural data, single-molecule approaches are also powerful to probe domain movements and complex assembly and lifetimes as well as disentangle the dynamics of the underlying processes both *in vivo* and *in vitro*.

## Conflict of interest statement

The authors declare no conflicts of interest.

## References and recommended reading

Papers of particular interest, published within the period of review, have been highlighted as:

• of special interest
•• of outstanding interest
